# CRISPR/Cas9-mediated overexpression of long non-coding RNA SRY-box transcription factor 21 antisense divergent transcript 1 regulates the proliferation of osteosarcoma by increasing the expression of mechanistic target of rapamycin kinase and Kruppel-like factor 4

**DOI:** 10.1080/21655979.2021.1995106

**Published:** 2022-03-02

**Authors:** W. Zhang, Q. Wang, H. Du, S. Jiang

**Affiliations:** a Health Management Center, Shandong Provincial Hospital Affiliated to Shandong First Medical University, Jinan, China; b Department of Human Resources, Shandong Provincial Hospital Affiliated to Shandong First Medical University, Jinan, China; c Shanghai Engineering Research Center of Nano-Biomaterials and Regenerative Medicine, College of Chemistry, Chemical Engineering and Biotechnology, Donghua University, Shanghai, China; d Department of Orthopedics, Shandong Provincial Hospital Affiliated to Shandong First Medical University, Jinan, China; e Medical Science and Technology Innovation Center, Shandong First Medical University & Shandong Academy of Medical Sciences, Jinan, China; f Department of Orthopedics, Shandong Provincial Hospital Affiliated to Shandong University, Jinan, China

**Keywords:** osteosarcoma, lncRNA SOX21-AS1, mTOR, KLF4, hsa-mir-7-5p, hsa-mir-145-5p

## Abstract

Osteosarcoma, derived from primitive mesenchymal cells, is the most common primary solid malignant tumor of bone. The cause of osteosarcoma remains unclear. In recent years, the role of non-coding sequences in regulating protein expression in tumors has been paid more and more attention, especially long non-coding RNA (lncRNA). We speculate that SRY-box transcription factor 21 antisense divergent transcript 1 (SOX21-AS1) can regulate the expression of the mechanistic target of rapamycin kinase (mTOR) and Kruppel-like factor 4 (KLF4) through sponging hsa-mir-7-5p and hsa-mir-145-5p. We knocked lncRNA SOX21-AS1 into the genome of 143B cells through CRISPR/Cas9, then screened out a monoclonal cell line. Detect the transcription level and protein expression level of the above-mentioned related genes, and cell proliferation. Then, ginsenoside Rg3 was added to culture the cell line knocked into lncRNA SOX21-AS1, and the expression levels of lncRNA SOX21-AS1, hsa-mir-7-5p, hsa-mir-145-5p, mTOR, and KLF4 were detected by RT-qPCR and Western blot. Cell proliferation method detects cell viability, explores the molecular mechanism of lncRNA SOX21-AS1 in osteosarcoma, and checks whether it can be used as a potential drug target for the treatment of osteosarcoma. Our results demonstrate that the overexpression of lncRNA SOX21-AS1 up-regulates mTOR and KLF4 by sponging hsa-mir-7-5p and hsa-mir-145-5p, and ultimately regulates the proliferation of osteosarcoma. It is proved that ginsenoside Rg3 can inhibit the cell proliferation of osteosarcoma by reducing the expression level of lncRNA SOX21-AS1. It provides an alternative for the treatment of osteosarcoma in the future.

## 1. Introduction

Osteosarcoma is a malignant tumor originating from mesenchymal stem cells[1]. Its morbidity and mortality rank first in bone tumors. Today, the pathogenesis of osteosarcoma is still unclear. The current treatment of osteosarcoma is still based on surgery combined with adjuvant chemotherapy. Therefore, current clinical treatments cannot effectively cure osteosarcoma[2]. With the continuous development of oncology research, gene-targeted therapy has become possible, bringing hope to overcome osteosarcoma. Gene targeted therapy is to intervene in abnormally expressed cancer-related genes in the development of osteosarcoma through molecular biological means, thereby inhibiting the malignant biological characteristics of the tumor, and achieving the goal of curing osteosarcoma. Therefore, exploring and discovering new molecular targets for osteosarcoma treatment will provide data support and a theoretical basis for gene targeted therapy.

The peak incidence of osteosarcoma is in adolescents who are 10–14 years old in the developmental stage. Juvenile osteosarcoma often appears in the metaphyses (75%) of long bones of the limbs, such as distal femur, proximal tibia, and proximal humerus. This also suggests that the pathogenesis of osteosarcoma is closely related to puberty hormone changes and bone growth[3]. Coincidentally, serine/threonine kinase 3 (AKT3) is a cell signaling regulator that responds to insulin and growth factors. It is involved in a variety of biological processes, including cell proliferation, differentiation, apoptosis, and tumorigenesis[4-6]. The AKT serine/threonine kinase can interact with Kruppel-like factor 4 (KLF4)[7] and mechanistic target of rapamycin kinase (mTOR)[8].

MTOR mediates cell responses to stresses, such as DNA damage and nutritional deficiencies. There have been thousands of papers on mTOR research, which have confirmed that it is a proto-oncogene[8-10]. KLF4 is thought to control the transition from G1 to S in the cell cycle after DNA damage by mediating the tumor suppressor gene p53, it is a tumor suppressor gene[11,12]. Over the past 10 years, the discovery and characterization of long non-coding RNA (lncRNA) with a length of more than 200 nucleotides has revealed the diversity of its regulatory role in tumors, and is considered as an important target for the treatment of human cancer. One of the roles of lncRNA is to sponge miRNAs as ceRNA to regulate the expression of related proteins[13]. If the expression levels of genes that are antagonistic to each other in the downstream genes regulated by the same lncRNA increase at the same time, then what will the final result of the regulation of this lncRNA be? Coincidentally, both mTOR and KLF4 are the target genes of hsa-mir-7-5p [14-16] and hsa-mir-145-5p [17,18]. More coincidentally, both hsa-mir-7-5p[19] and hsa-mir-145-5p[20] can be sponged by lncRNA SRY-box transcription factor 21 antisense divergent transcript 1 (SOX21-AS1). Therefore, we speculate that SOX21-AS1 can regulate the expression of mTOR and KLF4 through sponging hsa-mir-7-5p and hsa-mir-145-5p, thereby regulating the proliferation of osteosarcoma.

Therefore, we use CRISPR/Cas9 gene editing technology to knock lncRNA SOX21-AS1 into the “safe harbor” AAVS1 site, and then use RT-qPCR and western blot to detect the expression level of related genes, and use the cell proliferation method to detect the proliferation of osteosarcoma.

Ginseng is a herb native to Northeast Asia and is widely used in traditional medicine in East Asia. In recent years, more and more medicinal values of ginseng have been discovered and have attracted worldwide attention. Ginsenoside is regarded as the most important biologically active substance in ginseng[21]. In vivo and in vitro experiments confirmed that ginsenoside Rg3 can exert anti-tumor effects in a dose- and time-dependent manner[22,23]. In summary, we hypothesize that lncRNA SOX21-AS1 may enhance the proliferative properties of osteosarcoma by upregulating mTOR and KLF4, and verify whether ginsenoside Rg3 can inhibit osteosarcoma proliferation by targeting lncRNA SOX21-AS1, thus hopefully providing an alternative for future clinical treatment of osteosarcoma.

## 2. Materials and methods

### 2.1. Bioinformatics analysis

The data were acquired through TCGA database. The tools deepbase v3.0[24], UALCAN[25], RNAInter[26], UCSC Genome Browser[27], GEO DataSets, and GEPIA2[28] were used to analyze Gene Differential Expression, Gene Survival Analysis and gene interactions.

### 2.2. Plasmid

The PX459 vector (plasmid #62988) expressing the cas9 protein was obtained from Addgene. The gRNA sequence targeting AAVS1 intron 1 is 5ʹ-GGGGCCACTAGGGGACAGGAT-3ʹ[29]. As mentioned above, the SgRNA oligonucleotide was ordered (Sangon Biotech, Shanghai) and annealed into the PX459 vector digested with Bbs I. Then, we got the PX459-AAVS1-sgRNA plasmid. The Donor- SOX21-AS1 vector is kept by our laboratory (NCBI Reference Sequence: NR_046514.1). The vector was prepared by using Qiagen Endofree Plasmid Kit (Qiagen) and quantified by NanoDrop 2000 (Thermo Fisher).

### 2.3. Cell culture and transfection

The 143B cell line was cultured in DMEM (Invitrogen) supplemented with 10% fetal bovine serum (Sigma) and 1% penicillin–streptomycin (Hyclone, USA). 143B cells that reached 70%–80% confluence were co-transfected with sgRNA/Cas9-expressing PX459-AAVS1-sgRNA vector and Donor-SOX21-AS1 vector by lipofectamine 3000 (Thermo Fisher Scientific). Puromycin (0.5 μg/ml) was used to select transfected cells. The resulting cells are cloned and expanded by separating individual cells with limiting dilution method. Next, single-cell clones are picked and cultured in 96-well plates. The SOX21-AS1 knocked-in AAVS1 in a single isolated colony was detected by PCR, and then the amplicon was analyzed by Sanger sequencing (Sangon Biotech, Shanghai, China). Reporter gene Fluorescent protein is observed under 488 nm light wave. As mentioned in the previous study[30], a monoclonal cell line that successfully knocked into the SOX21-AS1 gene was cultured by adding ginsenoside Rg3.

## 2.4. qRT-PCR analysis

TRIzol reagent (Thermo Fisher Scientific, USA) was used to extract total RNA from cells. For specific experimental steps, please refer to this article-Quantitative RT-PCR[31]. Each experiment was repeated 3 times. The expression level of the target genes relative to β-actin was determined by the 2^−ΔΔCt^ method. The results are expressed as mean ± standard deviation. We use the two-tailed paired Student’s t-test for comparison. P < 0.05 is considered to be statistically significant.

The primer sequences were listed below:

sgRNA-F:CACCGGGGCCACTAGGGACAGGAT;

sgRNA-R: AAACATCCTGTCCCTAGTGGCCCC.

SOX21-AS1-F: ACGAGTAGGAGAGCCTCTCC;

SOX21-AS1-R: GCATACTCTCAGTCTGGGCTAGC.

mTOR-F: CAAAGCCGCCAAGGAGCTCC;

mTOR-R: ATGGCAAGACGGCCAATGGC.

KLF4-F: GGAGCTCTCCCACATGAAGCG;

KLF4-R: GGATAGGTGAAGCTGCAGGTGG.

KI Detection primer-F: CGAGAGATCTGGCAGCGGAG;

KI Detection primer-R: CGCTACAGGGCGCGTACTATGG.

### 2.5. Cell proliferation

143B cells were seeded into a 96-well cell culture plate at a density of 5000 cells per well. CCK-8 reagent (10 μl) was added to each well and incubated for 1 h. We use a microplate reader (Bio-Rad, Hercules, CA, USA) to measure the absorbance at 450 nm.

### 2.6. Western blot

The cell lysed supernatant was mixed with 4x SDS loading buffer and boiled for 5 min. The proteins were separated by SDS-PAGE and transferred to the PVDF membrane. After incubating with the primary antibody and the secondary antibody, we observe the protein bands with a chemiluminescent substrate working solution and image with X-ray film. We use Image J software to quantify the density of protein bands.

## 3. Results

Osteosarcoma has the highest morbidity and mortality rate among bone tumors. To date, the pathogenesis of osteosarcoma is still unclear, so current clinical treatment cannot effectively cure osteosarcoma. Therefore, it is urgent to explore and discover new molecular targets for osteosarcoma treatment. The discovery and characterization of long non-coding RNAs (lncRNAs) with a length of more than 200 nucleotides have revealed the diversity of their regulatory roles in tumors and are considered to be important targets for the treatment of human cancers. Therefore, in this study, the lncRNA SOX21-AS1, which is aberrantly highly expressed in sarcoma, was found by bioinformatics analysis. Hsa-mir-7-5p and hsa-mir-145-5p were also found to be sponged by lncRNA SOX21-AS1. Coincidentally, both mTOR and KLF4 are target genes of hsa-mir-7-5p and hsa-mir-145-5p, and both are also aberrantly highly expressed in sarcoma. Therefore, we speculate that SOX21-AS1 can regulate the expression of mTOR and KLF4 through sponging hsa-mir-7-5p and hsa-mir-145-5p, thereby regulating the proliferation of osteosarcoma. We use CRISPR/Cas9 gene editing technology to knock lncRNA SOX21-AS1 into the “safe harbor” AAVS1 site, and then use RT-qPCR and WB to detect the expression level of related genes, and use the CCK-8 method to detect the proliferation of osteosarcoma. Finally, we explored whether ginsenoside Rg3 could inhibit osteosarcoma by targeting lncRNA SOX21-AS1 to inhibit the proliferative properties of osteosarcoma cells.

### 3.1. KLF4 and mTOR are overexpressed in sarcoma(SARC)

**Figure 1.**
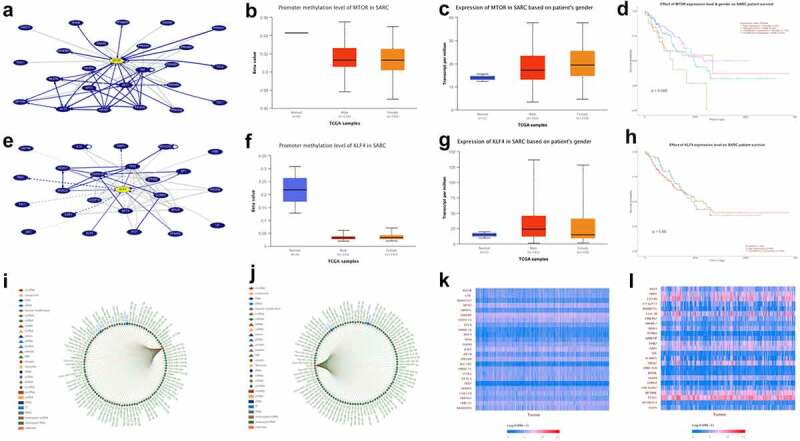
KLF4 and mTOR are overexpressed in SARC

The incidence of osteosarcoma is closely related to puberty hormone changes and bone growth. AKT3 is a cell signal regulator that responds to hormones, such as insulin and growth factors, and participates in a variety of biological processes, including cell proliferation, differentiation, apoptosis, and tumorigenesis [4-6]. Then, we use UCSC Genome Browser (http://genome.ucsc.edu/index.html) to analyze the network interacting with AKT3, and we find that both mTOR and KLF4 can interact with AKT3 (Figure 1(a,e)). There have been thousands of papers on mTOR research, which have confirmed that it is a proto-oncogene [8-10]. KLF4 is thought to control the transition from G1 to S in the cell cycle after DNA damage by mediating the tumor suppressor gene p53, it is a tumor suppressor gene [11,12]. Through TCGA database, we found that the promoter methylation level of mTOR and KLF4 decreased in sarcoma (Figure 1(b,f)). So, we speculate that their expression level in sarcoma should be elevated. Therefore, we further analyzed the expression of mTOR and KLF4 in patients with sarcoma of different genders, and the results did indeed increase in their expression (Figure 1(c,g)). KLF4 is a tumor suppressor gene, and mTOR is a proto-oncogene, so we speculate that overexpressed mTOR is not conducive to the prognosis of patients with sarcoma; the effect of KLF4 should be just the opposite. Then, we analyze the relationship between these two genes and the survival rate of sarcoma patients. Kaplan–Meier survival analysis shows that in sarcoma, the overall survival rate of patients with high mTOR expression is lower than that of patients with low mTOR expression, and the effect of KLF4 is really just the opposite (Figure 1(d,h)). Coincidentally, both mTOR and KLF4 are the target genes of hsa-mir-7-5p[14-16] and hsa-mir-145-5p[17,18] (Figure 1(i,j)). Genes positively related to mTOR and KLF4 in SARC indicate that these genes contain both proto-oncogene and tumor suppressor gene (Figure 1(k,l)), indicating that their regulation of tumors is an extremely complex network, which is the result of promoting tumor development and reducing patient survival by its combined effect.

(a) The network that interacts with mtor. (b) Promoter methylation level of mtor in sarcoma. (c) Expression of mtor in SARC based on patient’s gender. (d) Effect of mtor expression level on SARC patient survival. (e) The network that interacts with klf4. (f) Promoter methylation level of klf4 in sarcoma. (g) Expression of klf4 in SARC based on patient’s gender. (h) Effect of mtor expression level on SARC patient survival. (i) Genes that interact with mtor. (j) Genes that interact with klf4. (k) Heatmap of genes positively correlated with mtor in SARC (l) Heatmap of genes positively correlated with klf4 in SARC.

### 3.2. Highly expressed SOX21-AS1 significantly reduces the survival rate of SARC patients

**Figure 2.**
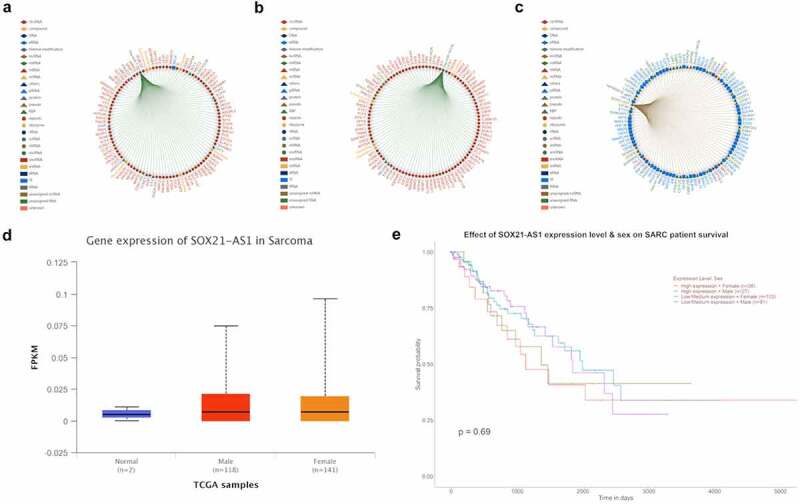
Highly expressed SOX21-AS1 significantly reduces the survival rate of SARC patients.

More coincidentally, both hsa-mir-7-5p[19] and hsa-mir-145-5p[20] can be sponged by lncRNA SOX21-AS1(Figure 2(a-c)), SOX21-AS1 can promote tumorigenesis[20], in the figure, the genes that interact with SOX21-AS1, hsa-mir-7-5p and hsa-mir-145-5p are all confirmed by previous studies. Therefore, we speculate that SOX21-AS1 can regulate the expression of mTOR and KLF4 through sponging hsa-mir-7-5p and hsa-mir-145-5p, thereby regulating the proliferation of osteosarcoma. It is worth noting that lncRNA SOX21-AS1 can sponge a lot of miRNAs. Among these miRNAs, hsa-mir-7-5p and hsa-mir-145-5p can also regulate many mRNAs and lncRNAs, so this is a very large and complex network. So if the expression of mTOR and KLF4 are regulated and increased by SOX21-AS1 at the same time, should the tumorigenesis be promoted or suppressed? The expression of SOX21-AS1 in sarcoma is slightly increased (Figure 2(d)), but Kaplan–Meier survival analysis shows that in sarcoma, the overall survival rate of patients with high SOX21-AS1 expression is lower than that of patients with low SOX21-AS1 expression (Figure 2(e)).

(a) Genes that interact with has-mir-7-5p. (b) Genes that interact with has-mir-145-5p. (c) Genes that interact with sox21-as1. (d) Expression of sox21-as1 in SARC. (e) Effect of sox21-as1 expression level on SARC patient survival.

### 3.3. Expression of SOX21-AS1, mTOR and KLF4 in pan cancers and co-expression with hsa-mir-7-5p and hsa-mir-145-5p in sarcomas

**Figure 3.**
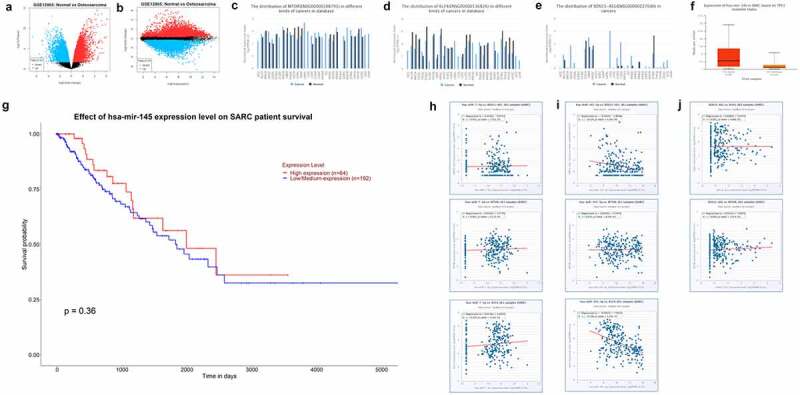
Expression of SOX21-AS1, mTOR and KLF4 in pan cancers and co-expression with hsa-mir-7-5p and hsa-mir-145-5p in sarcomas

We analyzed the differentially expressed genes in normal tissues and osteosarcoma through GEO DataSets database (GSE12865), mapped the volcanoes (Figure 3(a,b)) and found that mTOR and KLF4 were indeed significantly higher expressed in osteosarcoma. These are consistent with the results of the TCGA analysis above. Then, by collecting transcriptome sequencing results from previous investigators in different cancers, histograms of SOX21-AS1, mTOR and KLF4 expression in different cancers were plotted, which showed elevated expression of these three genes in most cancer tissues, especially in sarcoma tissues (Figure 3(c-e)). Then, we analyzed the expression of hsa-mir-145-5p in patients with osteosarcoma with TP53 mutation and its effect on patient survival (Figure 3(f,g)). TP53 is an oncogene and its mutation leads to increased proliferation of tumor cells. We found that hsa-mir-145-5p was increased in sarcoma with TP53 mutation. It should be that it has a synergistic effect with TP53, both have oncogenic properties, so when TP53 mutation cells enhance other oncogenic signaling pathways, the expression of hsa-mir-145-5p is increased, and high expression of hsa-mir-145-5p significantly enhances the survival rate of patients (high expression is 64 samples, low to medium expression is 192 samples), it was further demonstrated that hsa-mir-145-5p has cancer inhibitory effect. We then analyzed the relationship between hsa-mir-7-5p, hsa-mir-145-5p, SOX21-AS1, mTOR and KLF4 co-expression (more than 260 sample sizes) by transcriptome sequencing results of osteosarcoma from the database, and further prove the above conclusion (Figure 3(h-j)).

(a) Volcano plot of differentially expressed mRNA in gse12865. (b) Mean-difference plot of differentially expressed mRNA in gse12865. (c) Expression of mtor in different cancers with their corresponding normal tissues. (d) Expression of klf4 in different cancers with their corresponding normal tissues. (e) Expression of sox21-as1 in different cancers with their corresponding normal tissues. (f) Expression of hsa-mir-145-5p in sarcoma. (g) The effect of the expression levels of hsa-mir-145-5p on the survival of patients with sarcoma. (f) Co-expression of hsa-mir-7-5p with sox21-as1, mtor and klf4 in sarcoma. (i) Co-expression of hsa-mir-145-5p with sox21-as1, mtor and klf4 in sarcoma. (j) Co-expression of sox21-as1 with sox21-as1, mtor and klf4 in sarcoma.

### 3.4. Knock lncRNA SOX21-AS1 into 143B through CRISPR/cas9 gene editing technology

**Figure 4.**
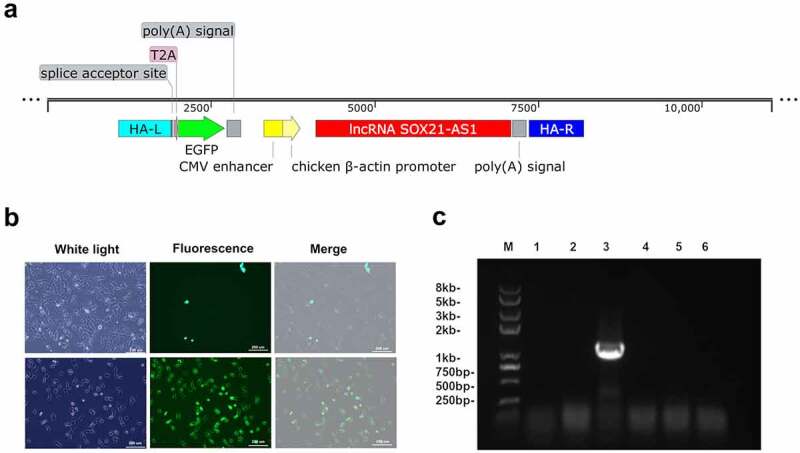
The lncRNA SOX21-AS1 was successfully knocked into 143B cells.

In order to verify our hypothesis, we used CRISPR/Cas9 to knock SOX21-AS1 into the genome of 143B cells[29]. After co-transfection of Donor-SOX21-AS1 and PX459-AAVS1-sgRNA plasmids, we observed a small amount of 143B cells expressing green fluorescent protein through fluorescence microscope (Figure 4(b)). Then, the monoclonal cell line was obtained by the limiting dilution method, and the KI-SOX21-AS1 143B monoclonal cell line was detected by PCR (Figure 4(c)). The screened KI-SOX21-AS1 143B cell line continued to be expanded and cultured (Figure 4(b)), and then sequenced to further confirm the successful SOX21-AS1 gene knock-in.

（a）Schematic diagram of the donor-sox21-as1. (ha-l/ha-r, homology arm; t2a, self-cleaving 2a peptide). (b) Fluorescence microscopy to detect the results of crispr/cas9 gene editing and ki-sox21-as1 monoclonal cells. (c) PCR detection of the Ki-sox21-as1 monoclonal 143b cell line.

### 3.5. SOX21-AS1 regulates the proliferation of osteosarcoma cells

**Figure 5.**
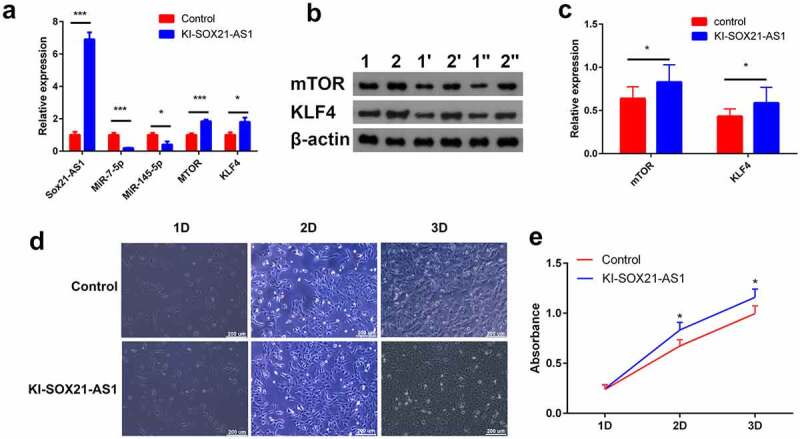
SOX21-AS1 up-regulates mTOR and KLF4 by sponging miR-7-5p and miR-145-5p, and ultimately regulates the proliferation of osteosarcoma cells.

The expression of SOX21-AS1 mRNA in the KI-SOX21-AS1 143B cell line was significantly increased (6.8 times). SOX21-AS1 as a sponge reduced the expression of miR-7-5p (5.18 times) and miR-145-5p (2.47 times), and finally indirectly increased the expression of mTOR (1.84 times) and KLF4 (1.79 times) (Figure 5(a)). WB results showed that the expression of mTOR (1.3 times) and KLF4 (1.35 times) was also significantly up-regulated at the protein level (Figure 5(b,c)). mTOR is a proto-oncogene and KLF4 is a tumor suppressor gene. Overexpression of SOX21-AS1 up-regulates the expression of both, so should 143B cell proliferation be promoted or inhibited? The results of cell proliferation experiments showed that SOX21-AS1 promoted the proliferation of 143B cells(Figure 5(d,e)). But KLF4 is a tumor suppressor gene, and its expression has also increased. Why do not KLF4 inhibit the proliferation of 143B cells? Therefore, we speculate that overexpressed SOX21-AS1 regulates many proto-oncogenes and tumor suppressor genes, mTOR and KLF4 are just two of these regulatory genes. The enhancement of cell proliferation is not only the sole effect of mTOR, but the result of combined action of many genes regulated by SOX21-AS1.

(a) The mRNA expression of sox21-as1, mtor, klf4, mir-7-5p and mir-145-5p. (b) Detect the protein expression of mtor and klf4 by WB (1,1ʹ,1„ represents the 143b cell line; 2,2ʹ,2„ represents the 143b cell line with sox21-as1 knocked-in). (c) Relative protein expression of mtor and klf4. (d) Detect cell proliferation with an inverted microscope. (e) The effect of sox21-as1 on proliferation in 143b cells was examined by cck-8 assay (control represents the 143b cell line. Ki-sox21-as1 represents the 143b cell line with sox21-as1 knocked-in).

### 3.6. Ginsenoside Rg3 inhibits the proliferation of osteosarcoma cells by inhibiting the expression of SOX21-AS1

**Figure 6.**
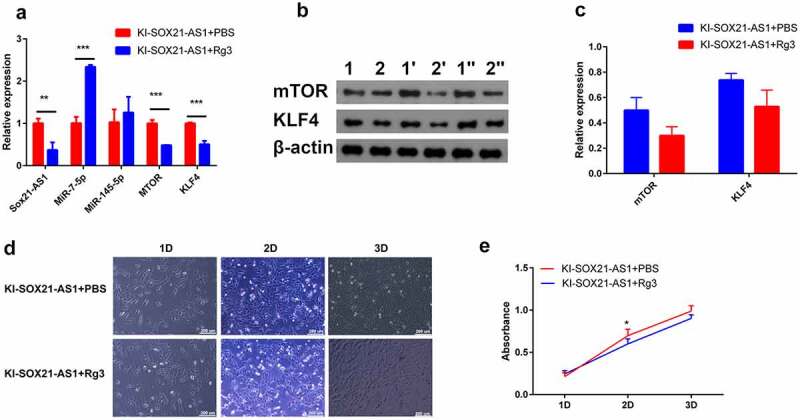
Ginsenoside Rg3 inhibits the proliferation of osteosarcoma cells by inhibiting the expression of SOX21-AS1.

To determine whether ginsenoside Rg3 affects the growth of 143B cells in culture, cell proliferation experiment, WB and RT-PCR were performed on 143B cells treated with 80 µM Rg3 for 48 hours[32]. Compared with control cells, the expression of SOX21-AS1 in 143B cells treated with ginsenoside Rg3 was significantly reduced (Figure 6(a)). Rg3 also significantly reduced the expression of mTOR and KLF4 (Figure 6(a-c)). Cell proliferation experiments have shown that Rg3 can inhibit the proliferation of 143B cells (Figure 6(d,e)). It provides an alternative for the treatment of osteosarcoma in the future. In conclusion, our results show that SOX21-AS1 can simultaneously up-regulate the expression of proto-oncogene mTOR and tumor suppressor gene KLF4, but its combined effect is to enhance the proliferation of 143B cells.

(a) The effect of ginsenoside rg3 on sox21-as1, mtor, klf4, mir-7-5p and mir-145-5p mRNA expression was detected by RT-PCR. (b) Detect the protein expression of mtor and klf4 by WB (1,1ʹ,1„ represents the 143b cell line; 2,2ʹ,2„ represents the 143b cell line with sox21-as1 knocked-in). (c) Relative protein expression of mtor and klf4. (d) Detect cell proliferation with an inverted microscope. (e) The effect of ginsenoside rg3 on proliferation in ki-sox21-as1 143b cells was examined by cck-8 assay (ki-sox21-as1 represents the 143b cell line with sox21-as1 knocked-in).

Therefore, we conclude that the regulation of lncRNA is not achieved through a single lncRNA/miRNA/mRNA axis, but through the comprehensive effect of a complex network. Perhaps, it can provide some reference value for studying the function of lncRNA in the future. Therefore, SOX21-AS1 may be a new indicator and potential therapeutic target for the progression and prognosis of osteosarcoma.

## 4. Discussion

Osteosarcoma is a malignant tumor originating from mesenchymal stem cells, its morbidity and mortality rank first among bone tumors. To this day, the pathogenesis of osteosarcoma is still unclear. Long non-coding RNA (lncRNA) is involved in the development of cancer. The lncRNA/miRNA/mRNA axis is very popular in the research of lncRNA. But metabolic regulation is a very complex network, some regulation has synergistic effects, some regulation has antagonistic effects. Therefore, if the expression levels of genes that are antagonistic to each other in the downstream genes regulated by the same lncRNA increase at the same time, then what will the final result of the regulation of this lncRNA be?

We have proved through bioinformatics that the expression of the proto-oncogene mTOR in sarcoma cells is increased, and its high expression leads to a low survival rate of patients. The expression of the tumor suppressor gene KLF4 in sarcoma cells is also increased, and its high expression leads to a high survival rate of patients. Coincidentally, both mTOR and KLF4 are the target genes of hsa-mir-7-5p[14-16] and hsa-mir-145-5p [17,18]. More coincidentally, both hsa-mir-7-5p[19] and hsa-mir-145-5p[20] can be sponged by lncRNA SOX21-AS1. Therefore, we speculate that SOX21-AS1 can regulate the expression of mTOR and KLF4 through sponging hsa-mir-7-5p and hsa-mir-145-5p, thereby regulating the proliferation of osteosarcoma.

Then, we successfully obtained the 143B cell line knocked into SOX21-AS1 through CRISPR/Cas9 gene editing technology. Our results prove that overexpression of lncRNA SOX21-AS1 up-regulates mTOR and KLF4 through sponging miR-7-5p and miR-145-5p, and ultimately promotes the proliferation of osteosarcoma. It is proved that Rg3 can inhibit the cell proliferation of osteosarcoma by reducing the expression level of SOX21-AS1. It provides an alternative for the treatment of osteosarcoma in the future.

In addition, the tumor suppressor gene KLF4 is overexpressed in sarcoma, which should be a negative feedback regulation of cell proliferation. Although the cell proliferation of sarcoma is still increased, this should be the result of negative feedback regulation, otherwise cell proliferation must be much higher than this result.

## 5. Conclusion

In this study, we demonstrated that the overexpression of lncRNA SOX21-AS1 upregulated mTOR and KLF4 through sponging hsa-mir-7-5p and hsa-mir-145-5p, and ultimately promoted osteosarcoma proliferation. And it was demonstrated that ginsenoside Rg3 could inhibit cell proliferation of osteosarcoma by reducing the expression level of lncRNA SOX21-AS1. It provides an option for the future treatment of osteosarcoma.
